# Storytelling in Pregnancy and Childbirth: An Integrative Review of the Literature

**DOI:** 10.1155/2022/8483777

**Published:** 2022-12-19

**Authors:** Zahra Mahdavi, Leila Amiri-Farahani, Sally Pezaro

**Affiliations:** ^1^Department of Reproductive Health and Midwifery, School of Nursing and Midwifery, Iran University of Medical Sciences, Tehran, Iran; ^2^Department of Reproductive Health and Midwifery, Nursing and Midwifery Care Research Center, School of Nursing and Midwifery, Iran University of Medical Sciences, Tehran, Iran; ^3^The Centre for Healthcare Research, Coventry University, UK

## Abstract

**Methods:**

An integrative review of the literature was conducted, including peer-reviewed articles published between 2001 and 2022. The following databases were used to search for relevant studies: PubMed, CINAHL, PsycINFO, Web of Science, Ovid, Google Scholar, ScienceDirect, Cochrane Library, Magiran, Irandoc, and SID. A process of thematic synthesis was used to make sense of the data extracted.

**Results:**

Whilst 21 studies were retrieved, only 12 were relevant and thus met the inclusion criteria set. Two themes were identified from our thematic synthesis: (1) effects of childbirth storytelling on the storyteller and (2) effects of childbirth storytelling on the listener of the story. Subthemes included “reducing fear of childbirth,” “transferring information and raising awareness in line with community culture,” and “adjusting expectations.”

**Conclusion:**

The use of storytelling can be used as an effective method in educational interventions during pregnancy and childbirth. Due to limited high-quality intervention studies in this field, future studies could usefully be more robustly designed and incorporate digital storytelling methods to inform future directions.

## 1. Introduction

Storytelling is a common and popular way of conveying events, ideas, and information not only in popular culture but also in the sciences [1, 2]. Significantly, conveying experiences and interactions between the narrator and the listener, stories can lead the listener to change their thoughts and behaviors [1]. Thus, storytelling can be an efficient way of conveying a message in a two-way process between both the sender and the narrator, allowing for a deeper understanding [2, 3]. In health, there is evidence that stories can facilitate the greater understanding of health messages and influence health-related behaviors [2]. When the story originates from real events and is in line with the real world, it can be particularly effective [3]. Consequently, this particular way of storytelling may be effective in healthcare scenarios, where experiences can be highly real, unique, and emotive in nature.

Childbirth is one such experience after which people who gave birth (most of whom are women) are known to transmit their stories and subsequent emotions to those around them [4]. This exchange of experiences can importantly reduce fear and anxieties, as a sense of power and courage from women who have given birth is transmitted to other women [5]. Such storytelling can also evoke a sense of agency [6] and encourage listeners to change their mental perceptions and perceptions based on their own perspectives and experiences [7]. This is particularly significant in the context of childbirth, where many women experiencing a fear of childbirth will request unnecessary interventions to avoid it [8].

There are several ways to use storytelling in interventions [9]. The variety in health education interventions include the use of written stories, storytelling videos, audio files of stories narrated by peers, and the use of mass media and virtual networks to convey a story to an audience [10]. In telling stories in a written way, the person imagines the story with his/her mentality, but in telling the story visually, he/she can direct the presentation of images [3]. The use of audio and video methods, due to the simultaneous transmission of nonverbal emotions and messages to the audience, can also be more effective in justifying the behavior change [11]. Comprehensive and sensory points are conveyed through video, at feature which does not exist with more traditional storytelling using written text or interviews [12]. Digital video storytelling, which is a combination of storytelling and technology in education, enables the sharing of information and increases the level of learning new content whilst activating the audience's mindset through storytelling [13]. Essentially, they change behavior by changing motivation [14]. Importantly, storytelling via digital media can lead to a more comprehensive understanding of content and experiences conveyed [15]. Therefore, this type of storytelling may also be most useful in improving experiences in childbirth.

Problematically, expressing experiences negatively can enable negative perceptions to develop. For example, hearing a negative experience of childbirth can lead to increased requests for unnecessary cesarean sections [16]. Whilst not ignoring the challenges of childbirth, in storytelling positive experiences, the end of the story is accompanied by a pleasant and satisfying feeling to convey a positive understanding of such challenges to the listener [3]. Thus, storytelling in relation to childbirth requires an empowering narrative.

Evidence demonstrates that changes in attitudes and beliefs have occurred more often as a result of hearing stories that arise from reality. For example, the impact of childbirth stories on people's minds has been shown to depend on a person's previous thoughts, culture, and the extent to which they are influenced by others [17]. The experience of giving birth also has an impact on society, and this effect has increased with the expansion of cybercommunities [18]. Yet receiving stories from different events can be very effective in developing richer contextual understandings in particular areas [19]. Thus, the use of storytelling may be particularly useful in the field of childbirth education.

A narrative of childbirth delivered as part of a prenatal education programme may not only be an innovative way to convey information but also a way to give meaning to complex information. Such methods can also lead to the shaping and changing of childbirth expectations, facilitate community support, and strengthen empathy along with positive communication [20]. Moreover, storytelling interventions in childbirth preparation classes can increase motivation [21]. Significantly, the telling of true childbirth stories can increase understandings and reduce fear of childbirth [22]. Considering the above, our objective was to synthesize existing literature in relation to storytelling in the context of childbirth to inform future childbirth education programmes, interventions, and research.

## 2. Methods

### 2.1. Design

This review of the literature was undertaken following the academic standards for conducting integrative literature reviews [23]. The updated Preferred Reporting Items for Systematic Reviews and Meta-Analyses (PRISMA) were adhered to in reporting [24].

### 2.2. Eligibility Criteria

Primary studies using either a quantitative, qualitative, or mixed-methods approach in English and Persian language were included. All included studies are related to storytelling in the context of childbirth. In order to garner more contemporary insights, articles were included if they were published anytime between 2001 and 2021. No restrictions were placed upon the outcomes for which data were sought in relation to the topic in question.

### 2.3. Information Sources

The following databases were used for searching: PubMed, CINAHL, PsycINFO, Web of Science, Ovid, Google Scholar, ScienceDirect, Cochrane Library, Magiran, Irandoc, and SID.

### 2.4. Search Strategy

Our search strategy was designed in partnership with librarians and used the following keywords: “delivery narrative,” “delivery storytelling,” “delivery scenario,” “peers,” “mode of delivery,” “perception of delivery,” “birth story,” and “personal narrative.” We selected the articles that were peer-reviewed. We also identified relevant studies cited by review articles.

### 2.5. Selection Process

**Figure 1:**
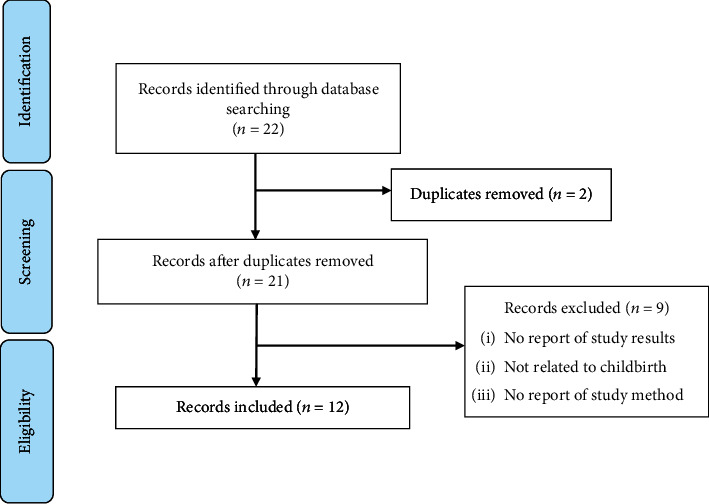
The articles included in the study.

Two duplicate records were removed by Z.M. from those identified via database searching (*n* = 22). Subsequently, remaining articles were screened against eligibility criteria in terms of relevance by Z.M. and L.A.F. at first individually and then via a series of academic discussions whereby the inclusion and exclusion of articles were agreed upon collectively. Articles which did not meet the inclusion criteria were excluded. Of the remaining 21 articles that were screened, nine articles were excluded as they did not report their results or study method and were not related to childbirth. Finally, 12 articles were included and synthesized (Figure 1).

### 2.6. Data Collection Process

The data were extracted by Z.M. manually.

### 2.7. Data Items

Extracted data included the study's title, country and city conducted in, participants' characteristics, intervention description, control or/and comparison groups, length of follow-up, the measure of outcome variables, and main results (Table 1).

### 2.8. Synthesis Methods

Data extracted from quantitative studies were analyzed via descriptive statistics and are summarized in Table 1. A summary of data extracted from qualitative studies is presented in Table 2. Our thematic synthesis of results is aligned with the integrative review method as set out by Cronin and George [32].

### 2.9. Quality Appraisal

The Mixed Methods Appraisal Tool (MMAT) outlined by Hong et al. [33] was applied individually by members of the team (Z.M. and L.A.F.). In applying the MMAT to both qualitative and quantitative studies, scores were given at ^∗^ if one criterion was met and ^∗∗∗∗∗^ if all five criterions were met.

## 3. Results

### 3.1. Study Selection

Duplicate articles (*n* = 2) were excluded. Subsequently, 21 studies were screened, and after reviewing the exclusion criteria, 9 were excluded due to an absence of storytelling content, being unrelated to childbirth, and having no reported methods used. Our selection process is detailed in Figure 1. Overall, 12 studies were included in our review. All selected studies were peer-reviewed and published between 2001 and 2022.

### 3.2. Study Characteristics

The use of storytelling in the field of childbirth has been explored in 3 quantitative studies and 8 qualitative studies. In respect of the qualitative articles, only two cases examined the effect of storytelling on the listener. Others focussed on the effects on the storyteller. Quantitative studies included clinical trials, which measured the effect of storytelling on listeners and/or readers.

### 3.3. Results of Individual Studies

Results of individual quantitative and qualitative studies included within this review are presented in Tables 1 and 2, respectively.

### 3.4. Results of Syntheses

Broadly, our synthesis of the literature identified two themes: (1) effects of childbirth storytelling on the storyteller and (2) effects of childbirth storytelling on the listener of the story. Subthemes included “reducing fear of childbirth,” “transferring information and raising awareness in line with community culture,” and “adjusting expectations.”

### 3.5. Theme One: Effects of Childbirth Storytelling on the Storyteller

Childbirth stories told by friends and close relatives are influential in shaping the experience of pregnancy and childbirth [27]. Yet the effect of storytelling on educational interventions during pregnancy and postpartum in storytelling was only identified in qualitative studies investigating why women want to retell their experiences and women's understanding of childbirth. In interviews with 22 women who had gone through childbirth, storytelling led to psychological relief, especially in those who had a traumatic or negative experience [30]. In this same study, the purpose of women in telling birth stories is to strengthen the motherly feeling by communicating with others and creating empowerment in women. Using maternity experiences to obtain women's perceptions of childbirth, one study examined the experiences of women who had endured a cesarean section via a content analysis of published childbirth stories [28]. In this study, women's experiences were found to be associated with feelings of shame resulting from childbirth. Nevertheless, those who engage in childbirth storytelling can be motivated to share their stories of childbirth to fulfill a sense of responsibility, normalize a variety of birth experiences, empower women, and seek validation [19]. Some are very much empowered in doing so [31], where storytelling allows for the reflective construction of meaning [31].

### 3.6. Theme Two: Effects of Childbirth Storytelling on the Listener of the Story

The consequences of using storytelling in the field of pregnancy and childbirth in studies with a quantitative and qualitative approach are as follows.

#### 3.6.1. Subtheme: Reducing Fear of Childbirth

Expressing individual experiences in a structured and orderly manner can lead to emotional reactions from others, which in turn reflect the impact of stories on others [17]. When women hear positive stories, they can feel empowered and their mental capacity for childbirth increases, and yet when they hear negative stories about childbirth, they can experience fear of childbirth [17]. To prepare women for childbirth in prenatal training to affect anxiety and fear of childbirth in one study, childbirth scenarios were used [26]. This study was performed as a clinical trial. In this study, one of the effects of training with simulated scenarios was a reduction in stress and fear of childbirth. In Rasoli et al.'s study to influence women's desire to choose vaginal childbirth, the use of birth scenarios in the intervention group also led to the desire of pregnant women to choose to birth their babies vaginally, influenced by reducing their fear of childbirth [25]. Interviews with 17 women who had experienced cesarean birth unearthed that one of the reasons for choosing this mode of birth was that they had previously heard stories of trauma in childbirth from relatives and friends [29]. Hearing positive childbirth experiences is effective in reducing the fear associated with progressive conditions, and negative experiences can increase maternal fear of childbirth and lead to a change in the choice of mode of childbirth in women [29]. Those engaging in childbirth storytelling are also motivated to listen to learn about birth [19].

#### 3.6.2. Subtheme: Transferring Information and Raising Awareness in Line with Community Culture

Peer-to-peer storytelling reinforced prelearned education and knowledge in pregnant women [17]. A qualitative study is aimed at explaining how knowledge related to pregnancy and vaginal childbirth is formed in prenatal sessions and the role of birth narrative in creating awareness and knowledge about childbirth was conducted [20]. Some (*n* = 10) of the total number of women (*n* = 25) who participated in the study gave birth and told the story of their childbirth to pregnant women. This increased their knowledge in relation to the realities of childbirth [20]. Through the qualitative analysis of childbirth stories told by 81 adolescents, storytelling was shown to convey knowledge and awareness about childbirth through the transmission of cultural norms [27]. Another qualitative study including interviews with women of two different generations during the postpartum period demonstrated that the increasing awareness and changing women's attitudes toward childbirth were in line with community culture [17]. Equally, storytelling can increase awareness related to labor in particular [26]. In these studies, the transfer of childbirth experiences as a way to transfer information combines culture and science related to childbirth and increases the awareness of pregnant women about childbirth.

#### 3.6.3. Subtheme: Adjusting Expectations

One of the benefits of childbirth stories was found to be the modification of the public's expectations of childbirth [17]. In Rasoli et al.'s study, the use of birth scenarios to influence the choice of mode of birth in the intervention group led to a change in the desire of pregnant women to choose to birth their babies vaginally rather than via cesarean section [25]. Indeed, the effect of hearing positive childbirth experiences in pregnancy can lead to more positive perceptions of childbirth [22]. Thus, adapting women's expectations of childbirth during pregnancy to develop a positive perception of childbirth to influence childbirth choices [25] may be key in the reduction of unnecessary cesarean sections.

### 3.7. Certainty of Evidence

The impact of childbirth storytelling has been used in limited studies. Due to the limited number of participants in the qualitative studies included, findings cannot be generalized. Furthermore, in the trials published, a lack of a clear method was observed. As such, confidence in the body of evidence for each outcome assessed is limited. The studies reported in the retrieved articles were of relatively high quality with MMAT scores ranging from ^∗∗^ to ^∗∗∗∗∗^.

## 4. Discussion

This integrative review of the literature has synthesized the existing literature in relation to storytelling in the context of childbirth. Ultimately, the telling of childbirth stories can adjust perceptions [17], shape experiences [27], reduce the fear and stress associated with pregnancy and childbirth [26], evoke psychological relief [30], increase women's tendency to choose vaginal childbirth via a reduction in fear of childbirth [25, 29], evoke empowerment [4], effectively convey information [20] through the transmission of cultural norms [27], allow for the reflective construction of meaning [31], and have a positive impact upon the perceptions of childbirth [22]. However, the sharing of childbirth stories can also evoke feelings of shame [28]. Nevertheless, those who engage in storytelling can be motivated to listen to learn about birth and be motivated to share their stories to fulfill a sense of responsibility, normalize a variety of birth experiences, empower women, and seek validation [19]. Consequently, storytelling may be usefully employed alongside other women's educational interventions to prepare for childbirth to increase the effectiveness of education.

Nevertheless, in qualitative studies, no specific implications have been considered, and due to the small number of interviewees who have heard the story, the implications are not generalizable to the community. A story established a connection between women and their shared backgrounds, and sharing stories regarding childbirth, as a seminal experience, should officially be taken into consideration in childbirth training programmes [25]. Narratives can be particularly effective when delivered via audio and video or by using longer texts. Narratives can also be effective when the content focuses on the detection and prevention of diseases [7]. Storytelling has also been used as an intervention in quantitative studies and clinical trials. These studies are somewhat limited as in one, storytelling was used alongside another intervention [25], and in another, only one outcome in the listener was examined [22]. Although experimental groups in narrative conditions were found to be more susceptible to persuasion than control groups, almost all of which received rational, factual arguments about health risks, the power of narrative persuasion must be further validated through comparisons with other frequently used message strategies in health communication such as fear, humor, frames, and cultural appeals [11]. One glaring gap in the extant narrative research is the impact of repeated exposures to stories. It is well known in social psychology and advertising that repetition provides individuals ample opportunities to engage in message processing [11]. The importance of looking at others' examples of digital resources was to guide the methodology for this phase regarding how to build them and what to include [14]. To make sense and in some way consolidate the differing models, it is necessary to base the approach on work that fitted with a humanistic, storytelling approach but also give a structured way to work and develop a resource.

## 5. Limitations, Future Research, and Implications of the Results for Practice

This review is limited in that only articles written in Persian and English were searched for and reviewed. The results of the studies should be interpreted with caution. Some related articles may not have been reviewed here. Thus, broader systematic reviews are required. Future studies will also require more scientific methods to assess the efficacy of childbirth storytelling as an intervention. Childbirth and pregnancy experiences are made available through virtual networks because of their availability and ease of use [30]. As such, it may also be useful to explore the efficacy of digital storytelling in this context. The studies included here show promise in storytelling improving childbirth education programmes and in reducing fear of childbirth and consequently the unnecessarily high rates of elective cesarean section. Thus, childbirth educators may usefully include childbirth storytelling in their programmes, though caution will be needed to address any feelings of shame which may arise in some cases.

## 6. Conclusion

The use of storytelling can be used as an effective method in educational interventions during pregnancy and childbirth. Due to limited high-quality intervention studies in this field, future studies could be more robustly designed and incorporate digital storytelling methods to inform future directions.

## Figures and Tables

**Table 1:** Quantitative studies evaluating the impact of storytelling.

Author, year, and location	Study groups	Intervention	Variable measure/scale	Results	MMAT score (^∗^ if one criterion is met; ^∗∗∗∗∗^ if all five criterions are met)
Rasoli et al., 2019, Iran [25]	Three groups consisted of two intervention groups (*n* = 74 each) and a control group (*n* = 75)	Education based on theory of planned behavior in two intervention groups and the written scenario of the birth story in one intervention group.	A researcher-made 80-item tool	The use of positive birth stories in education based on the theory of planned behavior has a greater impact on the behavioral intension of pregnant women and increases their desire to choose a vaginal delivery and leads to a decrease in the desire for cesarean delivery.	^∗∗∗∗^
Aydoğdu et al., 2020, Turkey [22]	Intervention group (*n* = 30) and control group (*n* = 30)	Three audio files of counterparts with a positive birth experience were provided to pregnant women in the intervention group.	25-item perception of birth (Fawcett, 1997)	The overall score of women's birth perception in the intervention group was higher than the control group. Birth perception is also generally influenced by the experiences that women hear about childbirth in and around their environment.	^∗∗^
Seong et al., 2020, South Korea [26]	Intervention group (*n* = 30) and control group (*n* = 30)	The training course consisted of 5 sessions per week. The educational content of the sessions was the awareness of pregnant women about labor and the teaching of relaxation and stress reduction techniques, as well as the association of similar conditions in different stages of labor and birth by expressing scenarios that were consistent with reality in each session.	20-item anxiety questionnaire (Spielberger, 1975), 26-item pregnancy-related stress (Ahn, 1985), knowledge of labor and birth (Kang, 1980), self-confidence concerning labor and birth (Lee, 2005), perception of labor and birth (Marut and Mercer, 1979)	The educational intervention was followed by increasing awareness and confidence related to labor and reducing stress related to pregnancy and shortening the time of labor stages.	^∗∗∗∗^

**Table 2:** Qualitative studies evaluating the impact of storytelling.

Author, year, and location	Behavioral consequences	Data collection method	Design of study	Participants	Results	MMAT score (^∗^ if one criterion is met; ^∗∗∗∗∗^ if all five criterions are met)
Kay et al., 2017, UK [17]	Receiving the expression of expectations and childbirth experience in the storytelling of childbirth of pregnant women from two different generations.	Interviews were collected by telephone and face to face and recorded as audio files. The duration of the interviews was 45 to 90 minutes	Interpretative hermeneutic phenomenological	20 pregnant women of two generations (10 women gave birth in 2013 and 10 women in 1980).	This study showed that birth stories are an important part of the birth perspective for pregnant women. Birth stories are cultural “productions” that convey different types of ideologies and belief systems.	^∗∗∗∗∗^
Carson et al., 2017, Canada [27]	Get the experience of women who have experienced pregnancy before work or school at a young age.	Face-to-face interview	Narrative analysis method was used to examine the stories told by adolescent mothers	Eighty-one pregnant women who became pregnant during adolescence recounted their postpartum experiences.	The telling of childbirth stories conveys cultural and social issues that affect the experience of childbirth. By telling the stories of childbirth, a connection is made between medical and social issues.	^∗∗∗∗∗^
Johnson et al., 2020, USA [19]	Investigating the cause and motivation of expressing childbirth experiences in the form of a story.	Interview collectively and again individually	Coding and qualitative analysis of data	Twenty-two women gave birth with 1 to 5 children and gave birth normally on average in the last 2.4 years.	Women were motivated to retell their childbirth experiences, to increase their sense of power, to validate their experience, and to feel responsible to their peers.	^∗∗∗∗∗^
Cobuzio, 2019 [28]	A review of childbirth stories in magazines and how to describe cesarean section in these stories.	—	Content analysis	The stories written on the two websites of mothers who shared their experiences were reviewed.	In women's birth stories, there was often a negative physical and emotional experience of cesarean birth. Most women experience childbirth with fear and shame.	^∗∗∗∗^
De Quattro, 2019, UK [20]	With the aim of explaining how knowledge is formed in pregnancy sessions and expressing the role of birth narrative in creating knowledge and understanding about childbirth.	The researcher attended the class in 6 sessions for 13 hours and recorded all the information and even recorded nonverbal observations	Content analysis	There were 25 people in the home birth group, of which 10 were pregnant women and 8 women were in the postpartum period, and 7 men were in the group. The center's training group consisted of 18 participants, including 10 primiparous pregnant women and 8 men.	Physiological childbirth education alone is not enough with health and hospital policies and approaches; rather, it is necessary to prepare pregnant women for childbirth by creating discourses. One way to create these discourses is to narrate the birth of other women who have experienced childbirth.	^∗∗∗∗∗^
Munro et al., 2009, USA [29]	Evaluation of the cause of cesarean birth without indication in primiparous women.	Face-to-face interview	Qualitative analysis	17 primiparous women with cesarean section without medical indication.	The experience of women having a cesarean section is influenced by cultural, social, and even historical factors, and all aspects must be considered in order to influence decision-making.	^∗∗∗∗∗^
McLachlan et al., 2016, UK [30]	Investigating the motivation of sharing maternity stories in virtual networks.	Mothers' website	Narrative analysis	Fifteen stories were selected from the virtual network and the website and analyzed.	Sharing the story of childbirth and childbirth experiences in cyberspace leads to healing the minds of mothers with traumatic childbirth experiences.	^∗∗∗∗∗^
MacLellan, 2019, UK [4]	To explore the influence of competing discourses upon woman's experience of birth in the UK.	Mothers' website	Structural and thematic analyses	20 birth stories written on the website were reviewed.	Reclaiming women's language for birth and working to create a new vocabulary encapsulating the experiences of birthing women will also present opportunities for the issue of birth and women's experiences of it to occupy greater political space with a confident and decisive voice.	^∗∗∗∗∗^
MacLellan, 2022 [31]	Study of the description of postpartum women from the experience of pregnancy and postpartum.	Mothers' website	Structural and thematic analyses	20 birth stories written on the website were reviewed.	Women described their bodies as a critical point for them to regenerate and regain their strength during childbirth. Recounting these experiences helped their sense of empowerment.	^∗∗∗∗∗^
